# Supplementary material for: UPF1 Inhibits Hepatocellular Carcinoma Growth through DUSP1/p53 Signal Pathway
Source: Biomedicines. 2022 Mar 29;10(4):793. doi: 10.3390/biomedicines10040793 (PMC9029930; doi:10.3390/biomedicines10040793)
Supplement: Supplementary file 1 [file biomedicines-10-00793-s001.zip › biomedicines-1664192-supplementary.pdf]

Figure S1

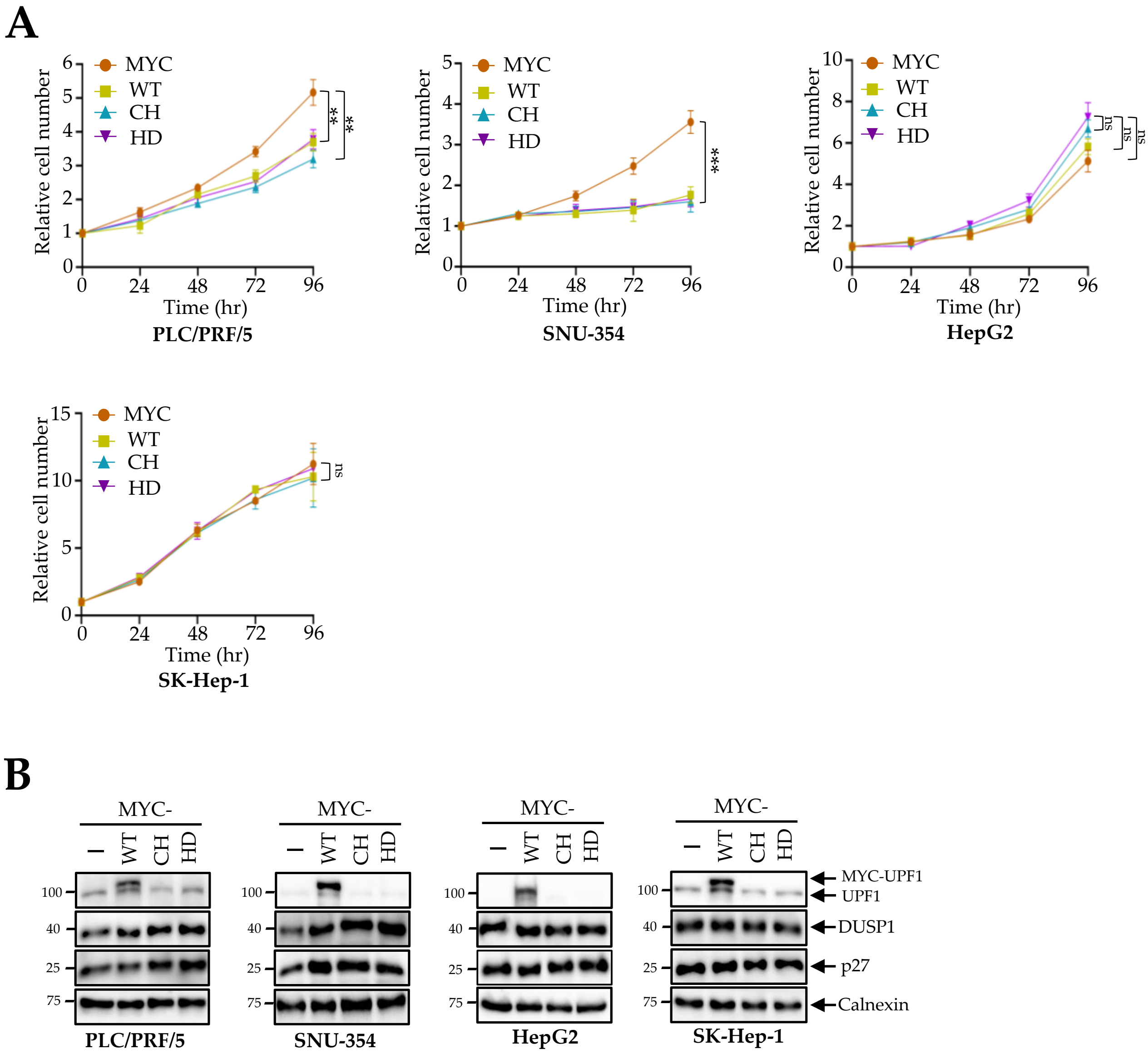

(A) PLC/PRF/5, SNU-354, HepG2 and SK-Hep-1 cells that were transfected with UPF1 variants or empty vector as the negative control. Cell growth was measured by counting the cells over a period of 96 hr. (B) Same as (A), however WB was performed to observe the indicated proteins.

Table S1. siRNA used in this study

siRNA name	Sense (5' to 3')
UPF1 CDS	CCAAGAUGCAGUUCCGCUCCAUU
UPF1 3'UTR	GCUUAGUCCAUCAGCAUCUUAUUCU

Table S2. Cloning primers used in this study

Primer name	Sequence (5' to 3')
UPF1-BamHI-F (1-1118)	CGGTACCCGGGGATCCATGGCATCAATGCAGAAGCTGATCTC
UPF1-BamHI-R (1-1118)	TATCGTCGACGGATCCTTAATACTGGGACAGCCCCGTCAC
UPF1-XhoI-F (1-244)	CGGTGACTAGCTCGAGATGGCATCAATGCAGAAGC
UPF1-NotI-R (1-244)	AGTTCTAGAGCGGCCGCTTACTTGACCAGCCAGGACAG
UPF1-XhoI-F (295-914)	GCGCTCGAGATGGCATCAATGCAGAAGC
UPF1-NotI-R (295-914)	ATATATGCGGCCGCTTAGCTGAACTGCATGAGG

qPCR primer name	Forward (5' to 3')	Reverse (3' to 5')
DUSP1	TCAAAGGAGGATACGAAGC	CCACCCTGATCGTAGAGT
Gl	TGCACGTGGATCCTGAGAACTTCA	ACCATTGTTCACAGGCAAGAGCAG
GPx1	CGGTTTCCCGTGCAATCAGTTCGG	TCACCATTCACCTCGCACTTCTCA
MUP	CTGATGGGGCTCTATG	TCCTGGTGAGAAGTCTCC
MAP3K14	GGCCCGTGTGTGTTGGAAGGG	GGTTCAGACATTGCAAGGGG
SMG5	CCCGAAGCAAAAGTCCTCCA	TCACGCAGCTTGTTCCTCAG
EZH2	GCTTCCTACATCGTAAGTGCAA	GCTCCCTCCAAATGCTGGTA
PEA15	ACATCCCCAGCGAAAAGAG	ACCATAGTGAGTAGGTCAGGAC
BAG1	AAGATGGTTGCCGGGTCATG	TGTTCTGCTCCACTGTGTCAC
TBL2	GCAGTCATTTACCACATGC	TATTGTTTCTGCTTCTTGGAT
GAPDH	CAAGATCATCAGCAATGCC	CTGTGGTCATGAGTCCTTCC

Table S3. qPCR primers used in this study
